# Picture perfect: A stimulus set of 225 pairs of matched clipart and photographic images normed by Mechanical Turk and laboratory participants

**DOI:** 10.3758/s13428-018-1028-5

**Published:** 2018-03-08

**Authors:** Raheleh Saryazdi, Julie Bannon, Agatha Rodrigues, Chris Klammer, Craig G. Chambers

**Affiliations:** Department of Psychology, University of Toronto, Toronto, Ontario Canada

**Keywords:** Stimulus set, Clipart images, Photographs, Norms, Visual iconicity, Mechanical Turk

## Abstract

The present study provides normative measures for a new stimulus set of images consisting of 225 everyday objects, each depicted both as a photograph and a matched clipart image generated directly from the photograph (450 images total). The clipart images preserve the same scale, shape, orientation, and general color features as the corresponding photographs. Various norms (modal name and verb agreement measures, picture–name agreement, familiarity, visual complexity, and image agreement) were collected separately for each image type and in two different contexts: online (using Mechanical Turk) and in the laboratory. We discuss similarities and differences in the normative measures according to both image type and experimental context. The full set of norms is provided in the supplemental materials.

**Electronic supplementary material:**

The online version of this article (10.3758/s13428-018-1028-5) contains supplementary material, which is available to authorized users.

Two-dimensional (2-D) images of individual objects are widely used as experimental stimuli in psychological research, and especially in work on attention, memory, and language. These stimuli are often most useful if they have been fully normed, either to ensure uniformity across the full set of items (e.g., in terms of recognizability, familiarity, name agreement, name frequency), or to allow researchers to explore how cognitive performance is related to variability in these measures. To date, the most widely used normed stimulus set has been the set of 260 line drawings developed by Snodgrass and Vanderwart (1980), as well as an updated colored version by Rossion and Pourtois (2004). However, advancements in digital photography and computer graphics, along with contemporary theoretical trends (e.g., embodied cognition) have led to increased interest in developing normed photographic stimuli (e.g., Adlington, Laws, & Gale, 2009; Brodeur, Dionne-Dostie, Montreuil, & Lepage, 2010; Moreno-Martínez & Montoro, 2012; Viggiano, Vannucci, & Righi, 2004), which provide more realistic depictions of real-world objects.

One consequence of the increasing availability of different stimulus sets is the potential for conducting comparative studies that explore whether and how certain cognitive processes might vary according to the type of 2-D image. Of particular relevance here is the issue of *visual iconicity*, namely differences in the degree to which images resemble the real-world objects they depict. For example, black-and-white line drawings, more realistic “clipart”-style images, and photographs can be understood as falling along a continuum ranging from less to more iconic depictions of the objects they represent in the world. A number of studies have explored various ways in which iconicity can influence aspects of perception and action as well as learning processes during the early years of development (e.g., Pierroutsakos & DeLoache, 2003; Simcock & DeLoache, 2006; Tare, Chiong, Ganea, & DeLoache, 2010; Troseth, Pierroutsakos, & DeLoache, 2004). For example, Pierroutsakos and DeLoache showed that 9-month-olds respond differently to 2-D images depending on the degree of iconicity (i.e., black-and-white line drawings, color line drawings, black-and-white photographs, and color photographs), such that successively more iconic images increased the manual behaviors children engage in when interacting with the corresponding 3-D object (e.g., attempting to drink from an image of a bottle). In other work, studies of concept acquisition in picture-book contexts have shown that children’s learning is improved with highly iconic images (i.e., photographs) as compared to less iconic images (i.e., line drawings; Ganea, Pickard, & DeLoache, 2008; Simcock & DeLoache, 2006; Tare et al., 2010).

In adults, processing benefits have sometimes been reported when comparatively more iconic 2-D images are used, as with Rossion and Pourtois’s (2004) addition of color and texture to the Snodgrass and Vanderwart (1980) images (although grayscale does not appear to confer any reliable benefit; Bonin, Méot, Laroche, Bugaiska, & Perret, 2017). Similar benefits have also been observed in studies that have compared more iconic images, such as photographs, with line drawings (e.g., Brodeur, O’Sullivan, & Crone, 2017; Brodie, Wallace, & Sharrat, 1991; Salmon, Matheson, & McMullen, 2014), or even when iconicity has been varied in smaller degrees (e.g., the ease of recognizing an object vs. its corresponding reflection in a mirror when given a photograph of a scene; Sareen, Ehinger, & Wolfe, 2015). In contrast, however, a number of behavioral and imaging studies have shown little or no effect of iconicity for 2-D images, with similar performance being found across different image types (e.g., Biederman & Ju, 1988; Kourtzi & Kanwisher, 2000; Snow, Skiba, Coleman, & Berryhill, 2014; Walther, Chai, Caddigan, Beck, & Fei-Fei, 2011). For example, Snow and colleagues examined recall and recognition performance for stimuli presented as real objects, colored photographs of those same objects, and black-and-white line drawing versions. Whereas recall and recognition performance was overall greater with 3-D objects, there were no differences between the two 2-D image types. The mixed pattern of overall findings can possibly be attributed to the differences in experimental paradigms as well as to the specific images used as visual stimuli.

One goal of the initiative described here was to facilitate future work in this area by providing a new stimulus set with closely matched items that vary systematically in their degree of iconicity and accompanying norms for each token. Our specific focus is a clear “missing link” in currently available normed stimulus sets (and many empirical studies), namely a high-quality and uniform clipart-style image that preserves relevant visual features of a corresponding photographic image. To date, normed stimulus sets of object photographs have focused primarily on the comparison of their photographic images with paired black-and-white line drawings or grayscale images (e.g., Brodeur et al., 2017; Moreno-Martínez & Montoro, 2012; O’Sullivan, Lepage, Bouras, Montreuil, & Broduer, 2012). For example, Moreno-Martínez and Montoro compared norms on their new photographic stimulus set with items from other normed photographic sets, as well as the norms from Snodgrass and Vanderwart’s original set of line drawings. In this case, the comparisons were based on the match in object type alone, with no control over the objects’ visual characteristics (e.g., contours, coloring, orientation). In contrast, the Bank of Standardized Stimulus set (BOSS; Brodeur et al., 2010; Brodeur, Guérard, & Bouras, 2014) contains corresponding matched grayscale versions of all photographic stimuli and black-and-white line drawings for a subset of the photographs. Separate norms for the different image types have also been collected, which can then be directly used in empirical investigations of how different image types affect cognitive processing (e.g., Brodeur et al., 2017). However, matched colored clipart-style images are not included in this stimulus set or, to our knowledge, in any other existing normed stimulus set that varies the degree of iconicity in images of individual objects.

We believe the development of a fully matched stimulus set involving photographs and clipart-style images has considerable value for addressing both methodological and theoretical questions. First, the “jump” in the degree of iconicity between photographs and line drawings or grayscale images in available image sets is considerable, particularly given the importance of color in object recognition (Bonin et al., 2017; Bramão, Reiss, Petersson, & Faísca, 2011; Price & Humphreys, 1989; Rossion & Pourtois, 2004; Tanaka, Weiskopf, & Williams, 2001; Wurm, Legge, Isenberg, & Luebker, 1993). Second, (colored) clipart images are arguably among the most widely used image types in certain subfields, such as in psycholinguistic work using the visual world paradigm. In the typical implementation of this paradigm, eye movements are recorded as listeners hear spoken instructions relating to depicted objects or scenes on a computer screen. The timing and pattern of eye movements provides fine-grained insights into various aspects of linguistic processing in real time. Although there are some exceptions, clipart has become the most common type of image used in these experiments (yet, perhaps ironically, the sharp increase in the use of this paradigm in the mid-1990s began with studies of real objects; see Tanenhaus, Spivey-Knowlton, Eberhard, & Sedivy, 1995).

For reasons of ecological validity, it would be informative to better understand the extent to which different patterns might occur when photographs are used instead of clipart in this paradigm. Although various processing phenomena have to date been replicated in recent work using photorealistic scenes preserving the spirit of earlier clipart “scenes” (Coco, Keller, & Malcolm, 2015; Staub, Abbott, & Bogartz, 2012), any observed differences would have been difficult to interpret given that the perceptual match between the two image types was not controlled. At the object level, for example, recognition is known to be influenced by factors such as shape (e.g., Biederman, 1987; Biederman & Ju, 1988; Sharma, Gupta, & Malik, 2012) and orientation (Bartram, 1976; Lawson & Humphreys, 1996), as well as by the earlier-mentioned influences of color. In addition, studies of eye movement behavior have demonstrated an optimal viewing position for object recognition, whereby initial eye fixations are programmed to land on the center of a target object from the viewer’s point of gaze (Foulsham & Kingstone, 2013; Henderson, 1993; van der Linden & Vitu, 2016). In studies in which multiple 2-D images are presented within the same display, fixations to various objects will be planned using parafoveal information. Differences in shape or orientation could therefore entail slightly different landing sites, potentially adding noise to measures of real-time processing.

On the theoretical side, matched clipart and photographic stimuli can be used to more effectively explore qualitative differences in the information that is evoked when viewing these types of images. For example, although a photograph of an object constitutes a veridical representation of a category exemplar at a given moment in time, clipart images are better understood as a kind of “constructed” representation that is normally intended to represent something more generic. Consistent with this distinction, developmental research has shown that 3-D toy objects lead caregivers to engage in more exemplar-related talk with children than do pictures, which instead encourage talk about abstract kinds. However, when the 3-D objects are put “on display” (preventing direct interaction; e.g., encased in a transparent plastic box), an increase in talk about abstract kinds is found (Gelman, Chesnick, & Waxman, 2005). Moreover, follow-up work indicated that the effects were not due simply to differences in the level of overall perceptual detail (Gelman, Waxman, & Kleinberg, 2008). These findings suggest that the symbolic status or representational intent of objects, as well as the cognitive stance that perceivers adopt when viewing objects, has an important influence on conceptualization processes. We believe additional exploration of the relationship between iconicity and conceptual processes would also benefit from a normed stimulus set comprised of photographs and closely matched clipart stimuli.

The present stimulus set aimed to meet this challenge by creating “clipart” images that are directly generated from object photographs, preserving general color features as well as type, shape, size, and orientation. To maximize the utility of these images, various normative measures were collected separately for each image type. We also examined whether and how normative measures may vary depending on the experimental context, namely between online and laboratory experiments. To achieve this, we recruited two commonly sampled participant pools: participants recruited online via Amazon’s Mechanical Turk (MTurk), and students tested on-site in a university laboratory (in-lab). The motivation for this stemmed from the fact that the ease and efficiency of using online services to recruit participants has led to an increased reliance on online crowdsourcing platforms such as MTurk for data collection, particularly in the social sciences. This has raised certain concerns among researchers about the generalizability of data derived from these samples. Some of these concerns relate to the expertise level of the participants (Chandler, Mueller, & Paolacci, 2014; Hauser & Schwarz, 2016). For example, because participants on crowdsourcing platforms are likely to take part in a greater number of studies than participants tested in laboratory studies, they may be less naïve to the purposes behind common experimental tasks or may approach tasks with a slightly different mindset. A related concern involves the amount of attention deployed to the task, although the evidence regarding this issue is mixed. For example, whereas Goodman, Cryder, and Cheema (2013) reported MTurk participants as being less attentive than students tested in the laboratory, a recent series of experiments by Hauser and Schwarz (2016) indicated that MTurk participants were more attentive than laboratory participants (see also Crump, McDonnell, & Gureckis, 2013). We therefore chose to collect separate norms from crowdsourced and laboratory participants, and examined the data for similarities or meaningful differences. This strategy was also motivated by the clear possibility that an image set in which the primary manipulation involved degree of visual detail might be rated differently by laboratory and crowdsourced participants, due to the greater likelihood of having smaller screens or lower screen resolution in the latter group. Researchers employing our stimulus set may therefore wish to draw on norms that are tailored to the testing context they are using.

In the remainder of this article, we describe the procedures used to collect norms for an image set consisting of closely matched photograph and clipart images. The normative measures included various name agreement measures, familiarity, visual complexity, and a rating of image agreement (sometimes referred to as *imageability*). We also included a verb generation task, in which participants were asked to provide a verb most related to the depicted image, providing measures relevant to psycholinguistic studies and studies of embodied cognition.

## Method

### Participants

The final sample consisted of 240 participants, tested in two different contexts. One group of participants was recruited from Amazon’s Mechanical Turk (*N* = 100, *M*_age_ = 42.48, *SD*_age_ = 11.21), restricted to US-based workers with an 80% or higher approval rating. The second group of participants were students attending the University of Toronto Mississauga (*N* = 140, *M*_age_ = 18.88, *SD*_age_ = 1.45). These participants received either partial course credit or monetary compensation for their time. The data from participants who learned English after the age of 5 or who repeated more than one norming experiment were excluded and replaced with new participants. No participants reported having color blindness. Different subgroups of participants were assigned to different tasks. Subgroup 1 completed the object naming task. Subgroup 2 completed the verb generation task. Subgroup 3 completed the picture–name agreement, familiarity, and visual complexity rating tasks. Finally, Subgroup 4 completed the image agreement task. All subgroups were tested both online and in the laboratory except Subgroup 4, which was only tested in the laboratory because control over the timing of stimulus presentation was required. Each of these subgroups was further divided into those who completed the clipart or photograph version of the norming experiments (with an equal number of participants in each version). Table 1 shows the breakdown of summary statistics by subgroups.

**Table 1:** Age of the participants in each testing group

	Photograph		Clipart	
	MTurk *M (SD)*	In-Lab *M (SD)*	MTurk *M (SD)*	In-Lab *M (SD)*
*Subgroup 1 (n = 60)*				
Object naming	35.67 (5.91)	18.4 (0.91)	40.4 (10.48)	19.07 (1.44)
*Subgroup 2 (n = 60)*				
Verb generation	46.87 (9.54)	19.27 (1.58)	42.73 (12.06)	19.13 (1.88)
*Subgroup 3 (n = 80)*				
Picture–name agreement, Familiarity, Visual complexity	45.8 (12.47)	18.55 (1.00)	42.35 (12.31)	18.6 (1.19)
*Subgroup 4 (n = 40)*				
Image agreement	—	19.55 (2.64)	—	19.10 (1.62)

### Stimulus set

**Fig. 1:**
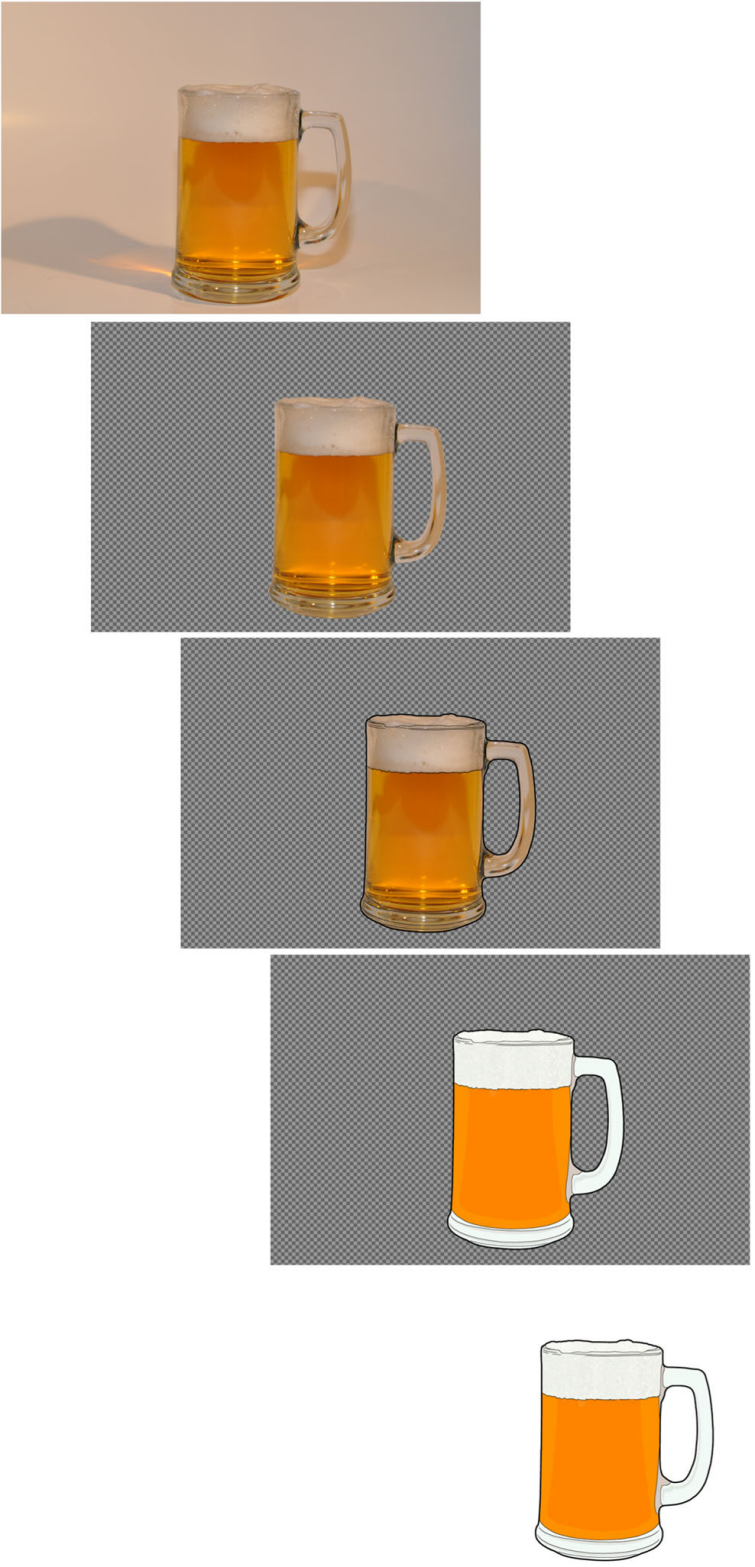
Example of photograph-to-clipart conversion

**Fig. 2:**
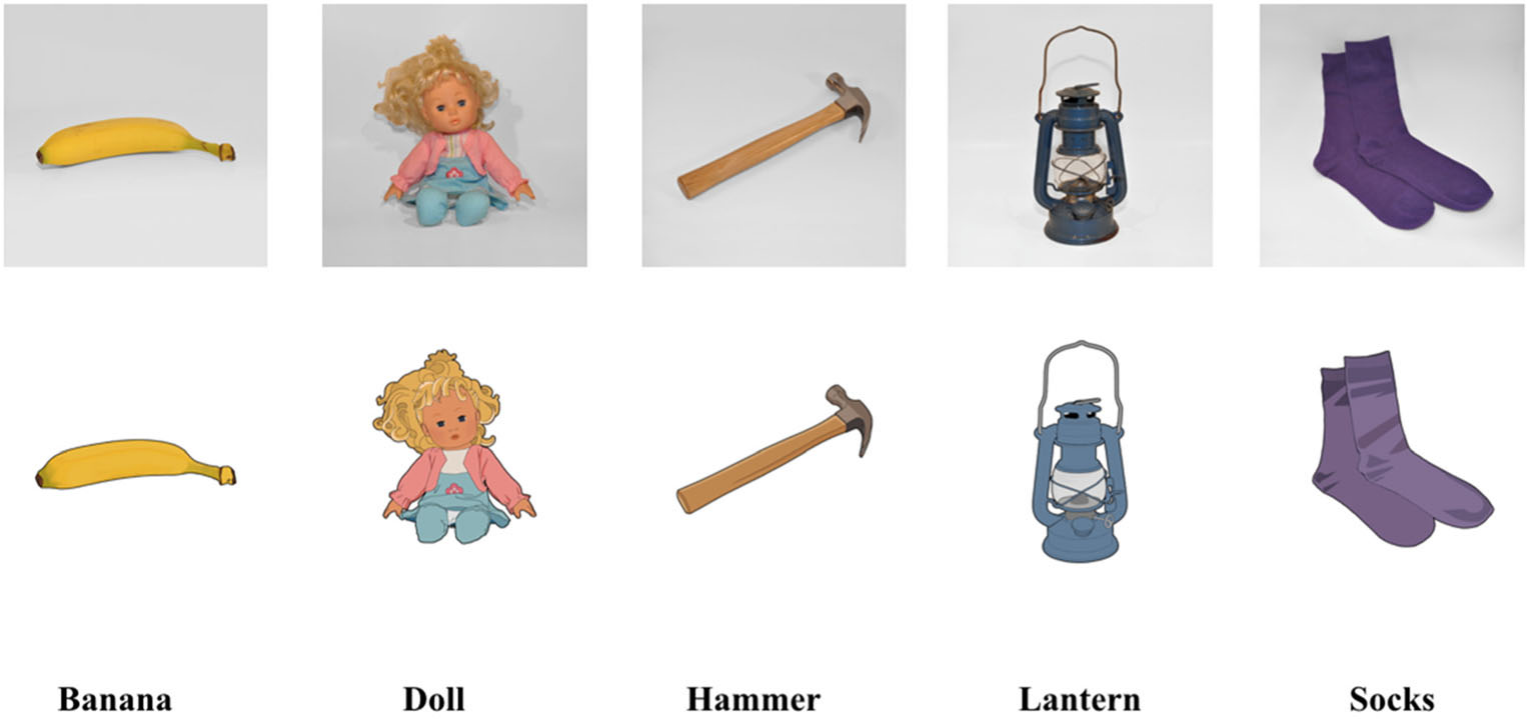
Sample image pairs

The present stimulus set includes 225 pairs of matched photographs and clipart images (450 total images). The stimulus set is composed of everyday real-world objects from a wide range of categories (see Table 2, using categories from Brodeur et al., 2010), with approximately 30% overlap with Snodgrass and Vanderwart’s (1980) objects. Every object was photographed with a Nikon D90 DSLR camera fitted with a Micro-Nikkor 40-mm f/2.8G zoom lens. Objects were photographed against a white background that involved both the supporting surface and the area behind the object. To ensure the highest degree of iconicity, shadows (if present) were retained in the photographs. However, the white background/surface was sometimes adjusted so it would be similar in shade across the set of photographic stimuli. The authors verified and approved the quality of each photographic image before proceeding to the next step (otherwise, new photographs were taken). Digital editing software (GIMP, Version 2.0, GIMP Development Team) was then used to create a clipart-style image corresponding to each photograph. Figure 1 illustrates an example of photograph-to-clipart conversion. All final clipart images had characteristics that were consistent with the general nature of clipart stimuli (e.g., defined black outline, white background, and uniform and bright colors; see the examples in Fig. 2). The full set of images and the full set of norms (supplemental materials) can be freely downloaded using a link from author C.C.’s laboratory website.

**Table 2:** Object categories

Category	Count	Category	Count
Food	37	Tool	11
Kitchen item	33	Sports	7
School/office supply	25	Music	5
Toy	20	Medical	5
Electronic	15	Jewel	4
Other	15	Decoration	4
Household article	14	Natural	2
Clothing	14	Furniture	2
Bathroom item	12

### General procedure

All norming tasks except the image agreement task were implemented and administered using the Qualtrics online survey tool (Qualtrics, Provo, UT) for both the MTurk and in-lab participants. The image agreement task was conducted using Experiment Builder software (SR Research, Ottawa, Canada) and was only presented to the in-lab participants, as described below. Each task was preceded by a language questionnaire used for screening participants, followed by an example trial explaining the nature of the task. Images were presented in random order and at a reduced size of 500 × 500 pixels to facilitate presentation on Qualtrics (the original images are 768 × 768 pixels). The instructions provided for the object naming task, picture–name agreement, familiarity, visual complexity, and image agreement were adapted from the original Snodgrass and Vanderwart (1980) study. In addition, we included a verb generation task similar to the object naming task in which participants were asked to generate a verb for a given image (e.g., where *throw*, *bounce*, or *roll* might be elicited for an image of a ball).

### Naming tasks

#### Object naming

Participants were instructed to type the common English label for each image. Every item was presented individually and accompanied by a text box. Participants provided their response at their own pace before moving to the next image. This task was conducted first in order to compute a name agreement score, which in turn allowed us to select the modal name to be used in the subsequent normative measures, as well as an *H* value capturing variability in the name assigned to an image.

#### Verb generation

Participants were asked to provide the verb (action word) they judged to be most strongly associated with the given image. Prior to this task, they were given two examples (car–*drive* and chair–*sit*) to ensure that they understood what was required. As before, participants provided their answer in a text box. As with object naming, a modal verb agreement score and an *H* value were calculated from the responses.

### Rating tasks

The picture–name agreement task, familiarity rating task, and visual complexity rating task were administered together as one experiment. Each image was accompanied by the most commonly given name determined by the object naming task unless the name with the highest incidence was deemed incorrect (occurring rarely; e.g., a bolt called a *screw*) in which case the correct name was used. The experiment was preceded by a detailed example whereby participants were given an explanation as to why someone would have chosen a particular scale for the exemplar (e.g., for the visual complexity rating, a hypothetical rater was said to respond “high” because the example image was judged to be visually intricate and detailed).

#### Picture–name agreement

Participants were asked to “Rate the degree of match between the depicted object and the label provided” using a 5-point Likert scale on which the lowest rating indicated the worst match and highest indicated the best match. To encourage thoughtful responses and the full use of the rating scale, the item set also included 25 additional images whose names were not a good match. Among these, some had incorrect names that were semantically associated with the depicted object (e.g., butterfly image–“grasshopper” label), whereas others were less related (e.g., traffic light image–“flag” label).

#### Familiarity

Participants were asked to rate how familiar they were with the object concept depicted in the image. They were given instructions to “Rate how often you interact with or think about the kind of object depicted in this image” on a 5-point Likert scale. They were informed that this was a test of familiarity and that selecting the lowest and highest values would indicate the item is unfamiliar or very familiar to them, respectively.

#### Visual complexity

Participants were asked to “Rate the two-dimensional image in terms of its level of intricacy or visual detail” using a 5-point Likert scale on which the lowest value represented a very visually simple representation and the highest value represented an extremely detailed representation.

#### Image agreement

As previously mentioned, this was the only task that was conducted in the laboratory using experimental control software (Experiment Builder; SR Research, Ontario, Canada) instead of the Qualtrics survey platform. Participants were provided the following instruction: “On each trial, a name of an object will appear briefly. Please picture that object in your mind. After a moment, an image will appear and you will be asked to rate how well this image matched the mental picture you formed.” The scale, which ranged from poor match to excellent match, was also described beforehand. Each trial began with a given word (the same as in the picture–name agreement task) being displayed for 3 s before disappearing, followed by a 2-s pause, after which the image appeared. At this point participants were presented with a question that asked “How well does this picture match the mental image you formed?” and were given a 5-point Likert scale for their response, as well as two additional options for “No Mental Image” and “Different Image.” Participants were instructed to select “No Mental Image” if they were unable to create a mental image for the object or they did not know the meaning of the given word, and to select “Different Image” if the image they were thinking of was a completely different kind of object than the one displayed. This task was only conducted with the in-lab participants as it required controlled timing in stimulus presentation, which is difficult to replicate online due to issues such as internet speed or participants momentarily leaving the task or being interrupted. Similar to picture-name agreement, this task also provides a measure of the fit between an image and a label. We chose to include both measures in order to be comprehensive and to reflect the measures provided in original study by Snodgrass and Vanderwart (1980).

## Results

Although our primary goal was to obtain the relevant norms for the stimulus set, we also examined whether the norms varied according to image type (photograph vs. clipart) and/or experimental context (MTurk vs. in-lab). All statistical analyses were performed using R open-source statistical software, Version 3.2.4 (R Core Team, 2016). Linear mixed-effect model analyses were conducted using the lme4 package, Version 1.1-11, and lmerTest, Version 2.0-30 (Bates, Mächler, Bolker, & Walker, 2015). We included image type, experimental context, and the corresponding interaction as fixed effects. Following Barr, Levy, Scheepers, and Tily (2013), we used a maximal random-effects structure that included an intercept for items and by-item random slopes for image type, experimental context, and their interaction, as well as an intercept term for participants (recall that the image type and experimental context manipulations were conducted between participants, and as such, no by-participants slope was included). For the naming tasks, the random intercept for participants was not included because the relevant measures involve aggregates of all participants’ responses. Any case in which a nonmaximal model was necessary for convergence is explicitly noted.

Table 3 provides correlation matrices to illustrate the degree of similarity in the normed measures across the two image types, collapsed across experimental contexts. After excluding the measures based on the same task (agreement and *H* scores), the most highly correlated measures involve picture–name agreement and image agreement, which is to be expected, given the similarity of the tasks (see also Snodgrass & Vanderwart, 1980). In addition, the table shows that the correlations are quite similar for both the photographs and the clipart stimuli. However, as would be expected, the magnitude of the relation between visual complexity and the other measures differs for the clipart versus photographic images.

**Table 3:** Correlation matrices

	Name–*H*	Verb Agreement (%)	Verb–*H*	Picture-Name Agreement	Familiarity	Visual Complexity	Image Agreement
*Photograph*							
Name agreement (%)	– .78^*^	.16^*^	– .16^*^	.44^*^	.25^*^	.02	.18^*^
Naming–*H* value		– .18^*^	.16^*^	– .50^*^	– .27^*^	.01	– .29^*^
Verb agreement (%)			– .94^*^	.11	.12	.09	.12
Verb–*H* value				– .07	– .11	– .06	– .09
Picture–name agreement					.27^*^	.02	.58^*^
Familiarity						– .13^*^	.11
Visual complexity							– .16^*^
*Clipart*							
Name agreement (%)	– .79^*^	.15^*^	– .15^*^	.38^*^	.34^*^	.004	.09
Naming–*H* value		– .18^*^	.17^*^	– .43^*^	– .34^*^	.06	– .20^*^
Verb agreement (%)			– .94^*^	.07	.13^*^	.15^*^	.02
Verb–*H* value				– .09	– .13	– .16^*^	– .02
Picture–name agreement					.24^*^	.28^*^	.54^*^
Familiarity						– .06	.04
Visual complexity							– .01

Norms are collapsed across the laboratory and Mechanical Turk participants. Asterisks denote significance at the .05 level.

Table 4 provides a by-group summary of the results from the various naming and rating tasks. (Item-wise norms split by image type are provided in the supplemental materials.) We have separated the results by the in-lab and MTurk groups, and also provide results averaged across the experimental context manipulation (bottom rows). The results are also separated by image type, with results for the clipart condition in the rightmost columns and the photograph condition in the leftmost columns. The correlations between the two image types are presented in the last column. The strong, positive correlations between the various photograph and clipart measures (all of which are large in magnitude by Cohen’s [1988] guidelines) suggest that the two image types are relatively comparable in terms of the collected norms. Nonetheless, there are some notable differences, described in the following sections.

**Table 4:** Summary statistics

	Photograph				Clipart				Correlation
	Mean	*SD*	Min	Max	Mean	*SD*	Min	Max	
*Mechanical Turk*									
Name agreement (%)	86	18	20	100	86	19	27	100	.77
Naming–*H* value	0.76	0.69	0	2.84	0.81	0.73	0	2.64	.80
Verb agreement (%)	59	24	7	100	54	23	7	100	.73
Verb–*H* value	1.53	0.74	0	3.24	1.81	0.79	0	3.32	.73
Picture–name agreement	4.87	0.20	3.20	5	4.57	0.24	3.40	4.9	.61
Familiarity	2.97	0.63	1.80	4.45	3.43	0.58	2.05	4.75	.90
Visual complexity	2.89	0.50	1.75	4.10	3.11	0.46	1.90	4.25	.73
*In-Lab*									
Name agreement (%)	84	21	13	100	82	21	13	100	.79
Naming–*H* value	0.94	0.79	0	3.32	0.89	0.79	0	3.11	.75
Verb agreement (%)	54	23	7	100	56	22	7	100	.76
Verb–*H* value	1.78	0.80	0	3.37	1.76	0.77	0	3.32	.75
Picture–name agreement	4.75	0.22	3.60	5	4.73	0.26	3.55	5	.72
Familiarity	3.35	0.71	1.75	4.90	3.56	0.75	1.95	5	.92
Visual complexity	3.10	0.48	2.10	4.45	3.55	0.48	2.15	4.75	.64
Image agreement^a^	3.94	0.58	2.11	5	4.10	0.57	2.33	4.95	.84
*In-Lab and Mechanical Turk*									
Name agreement (%)	85	18	20	100	84	19	23	100	.83
Naming–*H* value	0.85	0.68	0	2.78	0.85	0.71	0	2.74	.85
Verb agreement (%)	57	22	10	100	55	21	13	97	.84
Assoc. Verb–*H* value	1.66	0.71	0	3.18	1.78	0.71	0.18	3.28	.86
Picture–name agreement	4.81	0.18	3.80	5	4.65	0.23	3.48	4.95	.74
Familiarity	3.16	0.64	1.88	4.65	3.49	0.64	2.15	4.82	.95
Visual complexity	3	0.48	1.93	4.22	3.33	0.45	2.02	4.38	.73

^a^The image agreement task was conducted only in the laboratory

### Object naming

For each object, we calculated the frequency of the names provided by each group of participants. We began by combining instances of the names that were considered to be the same, including misspellings, abbreviations (e.g., *CD* and *compact disc*), plural marking (e.g., *egg* and *eggs*), and elaborations involving nominal modifiers (e.g., *book*, *red book*, and *red hardcover book*). In addition, if participants frequently named an object with a modifier that is central to defining the type of object, then the modifier was included in the modal name (e.g., *baby bottle* vs. *bottle*). All final decisions on how to combine instances of the names were agreed upon by the first two authors, and if necessary the opinion of a third author was used to arrive at the final decision. We report both the most frequent name and the second most frequent name used by each group. The latter is only included if it appeared in at least 10% of the responses given for the particular image type.

#### Percentage of modal name agreement

One of the goals in the use of naming norms is to determine the modal name for each depicted image (which is used in subsequent norming tasks such as picture–name agreement, etc.). In the majority of norming experiments, the modal name is based on the frequency of the names given by one group of participants. However, in the present study, four separate groups of participants (in-lab–photograph, in-lab–clipart, MTurk–photograph, MTurk–clipart) completed the same task, and sometimes differed in terms of their choices of highest-ranking names. Therefore, the final modal name selection was always based on the highest overall name given across groups when frequencies were collapsed. We explored potential differences across conditions in the percentages of responses in which the modal name was selected. As we discussed earlier, all analyses were conducted with a linear mixed-effect model using a maximal model structure (Barr et al., 2013), and the results are presented in Table 5. The only significant effect observed in the modal name agreement analysis was that of experimental context, whereby the percentage of name agreement was slightly lower among the in-lab participants (*M* = 83%) than among the MTurk participants (*M* = 86%).

**Table 5:** Summary of the results for linear mixed-effect analyses

Effect	Estimate	*SE*	*df*	*t*	*p*
*Name Agreement (%)*					
(Intercept)	84.68	1.17	224	72.12	<.001
Image Type	0.58	0.36	224	1.64	.103
Experimental Context	– 1.41	0.44	224	– 3.18	.002
Image Type × Context	0.35	0.25	224	1.38	.170
*Naming: H Value*					
(Intercept)	0.85	0.04	224	19.05	<.001
Image Type	– 0.001	0.01	224	– 0.08	.940
Experimental Context	0.07	0.02	224	4.32	<.001
Image Type × Context	0.03	0.01	224	2.37	.019
*Verb Agreement (%)*					
(Intercept)	55.92	1.37	224	40.83	<.001
Image Type	0.95	0.40	224	2.39	.018
Experimental Context	– 0.74	0.48	224	– 1.55	.122
Image Type × Context	– 1.70	0.39	224	– 4.38	<.001
*Associated Verb: H Value*					
(Intercept)	1.72	0.04	224	37.70	<.001
Image Type	– 0.06	0.01	224	– 5.10	<.001
Experimental Context	0.05	0.02	224	3.31	.001
Image Type × Context	0.07	0.01	224	5.42	<.001
*Picture–Name Agreement*					
(Intercept)	4.73	0.04	92	118.05	<.001
Image Type	0.08	0.04	77	2.05	.044
Experimental Context	0.01	0.04	78	0.27	.788
Image Type × Context	– 0.07	0.04	76	– 1.80	.076
*Familiarity*					
(Intercept)	3.32	0.09	118	36.52	<.001
Image Type	– 0.17	0.08	76	– 2.06	.043
Experimental Context	0.13	0.08	78	1.56	.124
Image Type × Context	0.06	0.08	76	0.78	.440
*Visual Complexity*					
(Intercept)	3.16	0.09	92	34.21	<.001
Image Type	– 0.17	0.09	78	– 1.89	.063
Experimental Context	0.16	0.09	76	1.82	.072
Image Type × Context	– 0.06	0.09	76	– 0.68	.496
*Image Agreement*					
(Intercept)	4.01	0.09	55	46.65	<.001
Image type	– 0.08	0.08	38	– 1.00	.324

Experimental Context: In-Lab = 1, MTurk = – 1; Image Type: Photo = 1, Clipart = – 1. Significance was tested with lmerTest using Satterthwaite approximations for the degrees of freedom

#### Naming: H value

Another use of naming data is to assess the variability in the number of names given for each item. The most commonly used measure to assess this variability is the *H* statistic, which accounts for both the number of alternative names, as well as the proportion with which alternative names occurred for each item. *H* is calculated with the following formula:

$$ H=\sum \limits_{i=1}^k{p}_i{\log}_2\left(\frac{1}{p_i}\right) $$

where *k* refers to the number of alternative names given per each object, and *p*_*i*_ refers to the proportion of participants selecting each name (Snodgrass & Vanderwart, 1980). An *H* value of 0 represents the highest agreement (no alternative names). Positive increases beyond the zero point reflect a correspondingly greater number of alternative names, and thus more variability. Although an analysis of *H* statistics is sometimes conducted after different instances of the same names are combined (e.g., with or without modifiers), we chose to assess variability on the raw naming data (combining only misspellings and plurality instances, and excluding “don’t know” responses), because we felt these data were more relevant for evaluating the possible effect of different experimental contexts and image types. For example, raters might provide comparatively more or less descriptive content when naming an object, depending on the image type or testing context.

The same linear mixed-effect model structure was used to examine the *H* values, except that the slope term for the interaction in the random effects was dropped because that model did not converge. Although the variability in object naming did not differ between the two image types, it did differ as a function of experimental context, such that the in-lab participants were more variable in naming objects than were the MTurk participants. However, this result was qualified by a significant Image Type × Experimental Context interaction (see Table 5). Follow-up tests revealed that group differences were found in both the photograph, *β* = 0.09, *SE* = 0.02, *t*(224) = 4.68, *p <* .001, and clipart, *β* = 0.04, *SE* = 0.02, *t*(224) = 2.20, *p* = .029, conditions, but the beta weights and means indicated that the interaction resulted from a larger difference in the photograph condition, in which the MTurk participants showed more consistency in responses (*M* = 0.76, *SD* = 0.69) than did the in-lab participants (*M* = 0.94, *SD* = 0.79). Note that these results are based on the raw naming data and thus do not suggest that the objects were not recognized to the same extent, but only that there seems to be more variability in the numbers and types of modifiers used for naming objects.

### Verb generation

This measure was included to provide psycholinguistic studies and studies of embodied cognition using visual stimuli with normative data on action verbs related to the depicted object. The normative verb associations used in the literature have often been based on corpus data (word–word associations), whereas the present study assessed word–image associations. We followed an analysis procedure similar to what was used for the object naming norms. Specifically, we report the frequencies of the highest and second-highest occurring verbs chosen for each item, but only when a given verb was selected by more than 10% of the overall participants. Verb counts were based on root forms (e.g., *hold*, therefore including *to hold*, *holds*, *hold a lot*, etc.). Additionally, we also calculated the *H* statistic for the verb data.

#### Percentage of modal verb agreement

A statistical analysis of the percentages of modal verb agreement revealed a significant effect of image type, with higher agreement scores for the photograph condition (*M* = 57%) than for the clipart condition (*M* = 55%). Although there was no main effect of experimental context, we did observe a significant interaction between image type and experimental context (see Table 5). Follow-up analyses revealed that the MTurk participants’ rate of agreement was higher in the photograph condition (*M* = 59%) than in the clipart condition (*M* = 54%), *β* = 2.66, *SE* = 0.59, *t*(224) = 4.54, *p* < .001. No significant difference was observed for the in-lab participants.

#### Verb: H value

An analysis of the *H* values for the associated verbs revealed a significant effect of image type, whereby there was more variability (higher *H* values) in verb responses when raters were presented with clipart images than with photographic images. We also observed more variability as a function of experimental context, with the in-lab participants providing more verbs per object than did the MTurk participants. These effects were qualified by a significant Image Type × Experimental Context interaction (see Table 5). Follow-up analyses revealed the MTurk participants to be slightly more consistent in the verbs they provided for the photograph condition (*M* = 1.53, *SD* = 0.74) than for the clipart condition (*M* = 1.81, *SD* = 0.79), *β* = – 0.14, *SE* = 0.02, *t*(224) = – 7.40, *p* < .001, but there were no differences between image types for the in-lab participants.

The patterns for verb generation were therefore very similar to those found for object naming, in which the highest agreement scores and consistency in naming were found when MTurk participants responded to photographic images. Finally, note that an informal comparison of agreement scores and *H* values across the object naming and verb generation tasks shows less agreement and more variability in verb generation. This is expected, because a variety of different actions can be associated with a particular object, whereas there are fewer obvious ways to name a common object.

### Picture–name agreement

Recall that this measure reflects participants’ judgments about how well the depicted image matched the provided label (which was the modal name selected in the object naming norms). The ratings were assessed using a scale on which a score of 5 reflected the strongest match between the modal name and the depicted image, and a score of 1 reflected the weakest match. The analysis revealed greater picture–name agreement for photographs (*M* = 4.81, *SD* = 0.18) than for clipart images (*M* = 4.65, *SD* = 0.23). Although the magnitude of this difference was small (i.e., 4% of the full scale), it suggests that participants considered the modal names to be a slightly better match for a particular object when it was depicted as a photograph rather than as a clipart image. One explanation for this pattern rests on the simple fact that photographs are more visually faithful to the real 3-D object they represent. The effect of experimental context and the Image Type × Experimental Context interaction did not reach significance.

### Familiarity

This measure assessed raters’ familiarity with each item in terms of how much they interacted or thought about the object. This analysis revealed that familiarity scores were significantly lower for photographs (*M* = 3.16, *SD* = 0.64) than for clipart images (*M* = 3.49, *SD* = 0.64). No other effects were significant. The small benefit observed for clipart images might have been due to the fact that they are typically understood to depict a general *kind* of object, whereas photographs reflect a specific *instance* of an object (which a participant will of course be less likely to have encountered). In point of fact, the language in the question explicitly asked participants how often they interacted with or thought about the *kind* of object being depicted. As before, however, it is important to stress that the magnitude of this difference was very small.

### Visual complexity

Visual complexity measures were also based on a 5-point scale. Although the analysis did not reach full significance for any of the fixed-effect or interaction terms, the results revealed a marginal effect whereby participants rated the photographic images (*M* = 3, *SD* = 0.48) as being less complex than the clipart images (*M* = 3.33, *SD* = 0.45). This was not expected, because clipart images, given their clear outlines and uniform coloring, seem likely to be perceived as visually less complex than photographs, which have more surface detail (e.g., shadows, texture). One possible explanation is that participants were judging our clipart images to be complex relative to other examples of clipart images with which they may have been more familiar. Another possible explanation is that clipart evokes the concept of a drawn image, causing participants to rate visual complexity from a “reproducibility” standpoint (whereby the act of creating a clipart image is complex in relation to the act of taking a photograph).

### Image agreement

As we mentioned earlier, the participants in this task were first given a name and asked to imagine the corresponding object. Then they were asked to decide how well a 2-D image presented shortly thereafter matched their imagined mental image of the object. The image agreement ratings for each item, as well as the number of times that participants reported they did not know the object or were thinking of a different kind of object, are included in the supplemental materials. Overall, there were very few instances in which these alternative options were selected. Whereas the total count of “Different Image” responses was slightly higher for clipart images (168) than for photographic images (143), the “No Mental Image” ratings were comparable between the clipart (64) and photographic (53) images. These values seemed to be more influenced by the particular item than by the type of image. For instance, almost half the participants chose “No Mental Image” for *squeegee* and “Different Image” for *mouse* (where “computer mouse” would have been a less ambiguous description for the depicted object but was not the modal name provided on the naming task). The “No Mental Image” and “Different Image” counts were excluded prior to the main analysis conducted on the image agreement scores. For this analysis, we applied the same model structure as before but did not include a term for experimental context or the interaction with this factor, because the task was only conducted with the in-lab participants. As is shown in Table 5, there was no reliable difference in image agreement scores for photographic (*M* = 3.94, *SD* = 0.58) versus clipart (*M* = 4.10, *SD* = 0.57) images.

## Discussion

We have described a new stimulus set of 2-D images consisting of 225 everyday objects, depicted both as a photograph and a carefully matched clipart-style image (450 images total). In all cases, the clipart images were custom-created directly from the photograph to ensure a close match in terms of relevant visual attributes (i.e., shape, orientation, size, and general color). The development of this unique stimulus set was motivated by the question of *visual iconicity*, namely the degree of realism inherent to a 2-D image. We argued in the introduction that the inclusion of colored clipart in a normed set of matched photographic images is important for several theoretical and methodological reasons and that, to our knowledge, there is no existing image bank of this sort.

The full set of images is accompanied by a range of norms that are traditionally provided for 2-D stimuli including object naming, picture-name agreement, familiarity, visual complexity, and image agreement. We also report normative data for a verb generation task in which participants were asked to provide the verb that was most strongly associated with the given image. Moreover, to further supplement the norms collected here, we provide additional measures from other sources. These include word frequencies for modal object names, using SUBTLEX-US measures limited to nouns only (Brysbaert & New, 2009; Brysbaert, New, & Keuleers, 2012), sense-specific noun frequencies from WordNet (Princeton University, 2010), and age-of-acquisition norms obtained from Morrison, Chappell, and Ellis (1997).

As we noted earlier, one question of interest was whether the type of pictorial depiction had any influence on the normative results. We also asked whether norms collected online (via Mechanical Turk) differed from those collected in the laboratory. Apart from enabling an empirical test of the effects of image type and experimental context, the provision of separate norms provides researchers with the option of selecting normative measures that correspond to the testing context they intend to use. Overall, we found that the normative results were quite similar across the two image types (photograph vs. clipart) and experimental contexts (MTurk vs. in-lab), with high correlations in the various normative measures. However, we did observe subtle differences for some of the norms collected. For example, photographs received higher picture-name agreement ratings than clipart images, possibly because their more detailed nature makes them more characteristic of the real-world objects they represent. In addition, clipart images were rated as higher than photographs in the familiarity norms, which asked participants to rate how often they interacted with or thought about the kind of object depicted in this image. We suggested this might be because clipart images are typically intended to represent a general kind of an object, whereas photographic images capture a particular instance of an object that participants may have not directly interacted with. Seeing as familiarity with kinds is no doubt greater than familiarity with a specific exemplar being depicted, the ratings for clipart stimuli may be correspondingly higher. However, as was also noted earlier, these differences were small in magnitude, and norms were otherwise very comparable across the two image types.

When contrasting the participants tested on Mechanical Turk and those tested in the laboratory, we found no differences on any of the numerical rating measures. We did, however, observe differences across experimental contexts in tasks in which participants were asked to provide the name of the depicted object or an associated verb. Specifically, MTurk participants were more consistent in their responses in comparison to the in-lab participants, particularly when presented with the photographic stimuli. One possibility is that this pattern arises because MTurk participants have substantial experience with naming tasks, and that photographic images provide some kind of additional familiarity benefit. This explanation is clearly speculative, however, and additional work would be required to fully understand the group differences.

Due to the complexity of the photograph-to-clipart conversion process, the current set is not as large as some recently developed stimulus sets (e.g., photographs: Brodeur et al., 2010; Brodeur et al., 2014; clipart: Duñabeitia et al., 2017), but nonetheless is likely to be sufficient for many experimental designs. Another potential limitation of the current set is that it does not include objects in certain categories (e.g., animals, furniture, body parts) that may be of relevance to some researchers. In addition, the data in the present study were collected using keyboard responses, and as such may or may not fully generalize to spoken language behavior. It is also possible that the norms assessed here may not fully generalize to speakers of English beyond North America, although this is a concern common to all initiatives of this type. Going forward, one possible extension will be to expand the norms for a broader range of subpopulations, such as older adults (e.g., Sirois, Kremin, & Cohen, 2006; Yoon et al., 2004) or speakers of different languages (e.g., Bonin, Guillemard-Tsaparina, & Méot, 2013; Brodeur et al., 2012; Duñabeitia et al., 2017; Kremin et al., 2003; Nishimoto, Ueda, Miyawaki, Une, & Takahashi, 2012; Shao & Stiegert, 2015). Indeed, given the substantial body of research examining visual iconicity and children’s understanding of the symbolic status of images, one obvious direction would be to extend the norms for the current stimulus set to children at various stages of development (see, e.g., Berman, Friedman, Hamberger, & Snodgrass, 1989; Cannard, Blaye, Scheuner, & Bonthoux, 2005; Cycowicz, Friedman, Rothstein, & Snodgrass, 1997). To facilitate this, we have included a number of items familiar to children (e.g., dolls, toy car, baby bottle, pacifier).

In conclusion, the new standardized stimulus set allows for a *comparison* of processing performance using photographs and closely matched clipart images. This comparison is facilitated by the degree of similarity across the image types in terms of surface-level features known to influence object recognition (i.e., color, shape, size, orientation), as well as by a range of relevant normative measures provided for each stimulus item. Furthermore, the results of our analyses indicate that the norms collected from online crowdsourcing services are for the most part comparable to those collected in a laboratory. Nonetheless, norms are provided separately for each image type and each experimental context, allowing researchers to draw on norms that are tailored to their experimental design.

### Author note

Funding for this research was provided by the Social Sciences and Humanities Research Council of Canada. The authors wish to thank Ben Bauer, Lena Donald, Yvette Hou, Sarah Macdonald, and Joanne Nuque for their assistance and advice, and the anonymous reviewers for their helpful comments and suggestions.

## Electronic Supplementary Material

ESM 1 (XLSX 159 kb).
